# Enamel and Dentin Caries Risk Factors of Adolescents in the Context of the International Caries Detection and Assessment System (ICDAS): A Longitudinal Study

**DOI:** 10.3389/fped.2020.00419

**Published:** 2020-07-28

**Authors:** Ketian Wang, Liangyue Pang, Cancan Fan, Tianqiang Cui, Lixia Yu, Huancai Lin

**Affiliations:** ^1^Department of Preventive Dentistry, Guanghua School of Stomatology, Hospital of Stomatology, Sun Yat-sen University, Guangzhou, China; ^2^Guangdong Provincial Key Laboratory of Stomatology, Sun Yat-sen University, Guangzhou, China; ^3^Foshan Stomatology Hospital, Foshan, School of Stomatology and Medicine, Foshan University, Foshan, China

**Keywords:** caries, ICDAS, ZINB, adolescent, epidemic

## Abstract

**Objective:** The objective of this study was to identify risk factors for enamel and dentin caries in adolescents.

**Method:** This 1-year longitudinal study was conducted in 2018 and 2019; 13- to 14-year-old adolescents were recruited. The merged International Caries Detection and Assessment System (ICDAS) was used to identify caries. The relationships between the caries increment and variables were analyzed with a zero-inflated negative binomial (ZINB) regression model.

**Results:** A total of 1,016 participants completed the assessment. The ZINB analysis found that individuals with caries at baseline were more likely to develop new dentin caries. Females, or individuals who had a high cariostat score had an increased likelihood of having a high ΔD4-6MFT score. Among the caries-free adolescents at baseline, females, or individuals who consumed snacks once or more than once a day were more likely to develop caries. Individuals from one-child families, who used fluoride toothpaste, and who had a high saliva buffering capability (pH≥4.25) had an increased likelihood of a low ΔD1-6MFT score.

**Conclusion:** The results suggest that there are some specific risk factors of initiating of enamel caries in adolescents, including the frequency of snack consumption, sex, saliva buffering capability, fluoride toothpaste usage and belonging to a one-child family. In all adolescents, most of whom have enamel caries, the dentin caries risk factors were past caries experience, cariostat score and sex.

## Introduction

Although preventive strategies have been implemented for decades, dental caries remain a major public health problem, especially in developing countries (1). Adolescence is a period in which the risk for dental caries remains especially high (2). The global caries prevalence was approximately 50% among 12–15-year-old adolescents and over 70% among 17-year-old adolescents (3). In China, the prevalence of caries among 12-year-old children has increased from 28.9 to 38.5% in the last decade (4, 5). These numbers have raised concerns about caries experience among adolescents. Adolescence is a vital life stage in which children begin to develop self-performed oral health habits instead of relying solely on parental supervision (6). Dental caries may lead to pain and swelling and affect early permanent dentition development in adolescents, potentially drastically reducing their quality of life (7). Therefore, it is important to adopt some intervention measures for the early prevention of caries among teenagers.

Caries risk assessment is known to be an essential element in the planning of preventive and therapeutic strategies (8). As one of the most common chronic, infectious and multifactor diseases among children and adults (9), dental caries have a complicated etiology model consisting of demographic factors, socioeconomic factors, oral health-related behaviors, and biological factors (10). These factors have been applied in some caries assessment systems (11, 12); however, few were targeted at adolescents, and the caries phenotypes were not distinguished in these assessment systems. Some cross-sectional studies have been performed to investigate the risk indicators for dental caries among Chinese adolescents (13, 14), but there is a lack of evidence of the risk factors according to longitudinal studies.

Due to (1) the high increasing caries rate, (2) critical stage for self-behavior formation, and (3) unique social and psychological needs (15), the primary objective of this study was to investigate the caries risk factors in adolescents in South China by conducting a 1-year longitudinal study, specifying the risk factors without the effect of past caries experience.

## Methods

### Ethical Approval

The study was approved by the Ethical Review Committee of Guanghua School of Stomatology, Sun Yat-Sen University (ERC-[2018]01). Written informed consent was obtained from each participant's guardian before the baseline and follow-up assessments. A verbal assent was obtained from each of the participants.

### Subjects

The baseline data were collected from March to April 2018 in Foshan. Foshan is a medium-sized city in Guangdong Province in southern China, with a population of 7.6 million. The gross domestic product (GDP) was CNY 124,324 (USD 18,018) per capita in 2017 (16). The water fluoride concentration is 0.16 mg/L (17).

In total, twenty independent variables were investigated in this study. PASS 11.0.7 (NCSS, LLC) was applied to calculate the sample size. The Poisson regression model was used. The significance level was set at 0.05, with a power of 0.8, assuming a 20% non-response rate; thus, the total sample size was calculated as at least 953 participants.

A multistage cluster sampling technique was employed. The size for each cluster was calculated by the following formula (18):

$$\begin{array}{l} r=\frac{Z^{2}VR}{{△}^{2}R+Z^{2}V} \end{array}$$

The calculated sample size (*R*) was 953, with a standard error set at 8% (Δ = 8%^*^2) and a confidence interval of 95% (Z = 1.96). According to the data from the 4th National Oral Health Survey in China, the variation coefficient (*V*) should be 1.53 (4). Hence, the cluster size should be 186 and the cluster number was 6 (953/186). Among 39 middle schools in Foshan in 2018, six of them were randomly sampled. Four grade seven classes were randomly sampled from each of the 6 schools as the class size was ~45 in Foshan schools. All the students in these classes whose parents were Han Chinese and permanent residents in Foshan were recruited for this study. Those who reported systematic illness or antibiotic use in the preceding 2 weeks were excluded.

For the longitudinal evaluation at the 1-year follow-up (2019), all adolescents who completed the assessments at baseline were invited to participate in a follow-up assessment.

### Oral Health Questionnaires

Detailed information was obtained through a structured questionnaire completed by the adolescents with the guidance of their guardians. The questionnaire was mainly designed with reference to the Fisher-Owens conceptual model of influence on children's oral health (19). It consisted of three parts, as follows:

1. Demographic information: sex, age, residence, whether the child is an only child in his/her family and his/her primary caregiver;
2. Socioeconomic information: family income, caregivers' education levels, and whether they have dental insurance; and
3. Oral health-related behaviors: tooth brushing frequency, flossing habits, toothpaste containing fluoride or not, frequency of snack consumption, frequency of sweet drink consumption, and dental attendance experience.

The compulsory education is 9 years in China; thus, “9 years” was set as a threshold to distinguish the education of the caregivers in the variable “education of caregiver.” As to the categories in the variable “household monthly incoming,” according to the Foshan government, households with the lowest 20% annual average household income are classified into the low income group, while households with the highest 20% annual average household income are classified into the high income group (16). In 2017, the thresholds were 35,339.38 CNY (~3,000 CNY per month) for the low income group and 85,774.21 CNY (~7,000 CNY per month) for the high income group in Foshan (20).

### Dental Examinations

**Figure 1:**
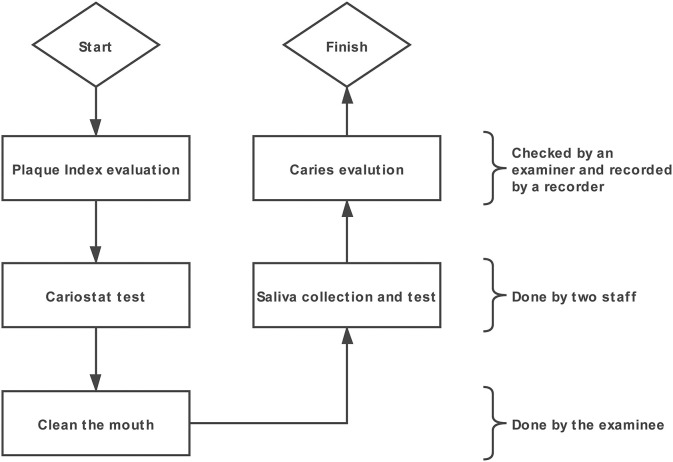
The procedure for dental examination and other tests.

Dental examinations were conducted under a portable light by three calibrated examiners, each of whom equipped with one recorder. A community periodontal index (CPI) explorer, a dental mirror sterile cotton swabs and compressed air were used during the dental examination. The procedure for all tests are shown in Figure 1. The Plaque Index was checked first. The Plaque Index (PlI) was recorded according to the Silness and Löe scale (21). After the teeth were brushed by the adolescent him/herself, the caries were examined. The merged International Caries Detection and Assessment System (ICDAS) criteria were used (22). All tooth surfaces were first examined with a wet surface and then re-examined after the teeth were dried with compressed air. Sterile cotton swabs were used during caries examination when debris remained on tooth surfaces. Teeth or surfaces filled and teeth or surfaces missing due to caries (extractions due to orthodontics or other reasons were not included, and a verbal confirmation was obtained from the examinee when unclear).

Three examiners attended a 12-h e-learning course on ICDAS before the study. To calculate the consistency of the inter-examiner reliability, 105 subjects were randomly chosen during the oral examination; they were re-examined by another examiner immediately after being examined by the first examiner (35 examinees from A to B, 35 examinees from B to C, and 35 examinees from C to A).

Decayed teeth were recorded as enamel caries (coded 1–3 in the ICDAS system, D1–3) or dentin caries (coded 4–6 in the ICDAS system, D4–6) (23). Filled surfaces and missing teeth due to caries (the reasons for missing teeth were obtained from the questionnaire) were also recorded (24).

The same three examiners who performed the baseline examinations performed the 1-year follow-up examinations. As in the baseline examinations, the caries were recorded as enamel or dentin caries according to the merged ICDAS. Filled surfaces and missing teeth due to caries were also recorded.

Similar to the baseline assessment, 105 subjects were randomly re-examined to calculate Cohen's kappa.

### Plaque and Saliva Samples

Plaque samples were collected using the sterile swab included in the cariostat kit (GangDa Medical Technology Co. Ltd., Beijing, China). According to the cariostat kit instructions, the examiners scrubbed the buccal surfaces of the maxillary molars and mandibular incisors 3–5 times and immersed the swab into the culture medium ampule. The samples were incubated at 37°C for 48 h. The color of the culture medium was compared with the reference color on the color chart supplied with the cariostat kit. A score of “0” designates the lowest acidogenecity of oral microorganisms, while “3.0” represents the highest acidogenecity of oral microorganisms (25).

Whole saliva was collected after the students had rinsed their mouths with tap water. Unstimulated saliva was collected for 15 min. Students were asked to spit the saliva through a funnel into a scaled tube every 3 min. Then, the unstimulated saliva flow rate (ml/min) was calculated. The flow rate was recorded into “abnormal (<0.25)” or “normal (≥0.25)” (26).

The saliva buffering capability was measured according to the Ericsson method (27). One milliliter of saliva was added to 3 ml of 3.3 mmol HCl within 5 min after collection and then allowed to stand for 20 min to remove CO_2_. The final pH of the saliva was evaluated by an electrical pH meter. The buffering capability was recorded into “low” (<3.5), “moderate” (3.5–4.25) or “high” (≥4.25) (28).

### Statistical Analysis

The data were analyzed by R 3.6.1 statistical software. Individuals with missing data were excluded from the analysis. The D1-3T/S, D4-6T/S, teeth or surfaces filled (FT/S) and teeth or surfaces missing due to caries (MT/S) were calculated both at baseline and at the 1-year follow-up. The following indices were calculated:

$$\begin{array}{l} \Delta \text{D1}-\text{6MFT}=\text{(D4-6Tfollow-up}-\text{D4-6Tbaseline)}\\ \;+\text{(D1}-\text{3Tfollow}-\text{up}-\text{D1}-\text{3Tbaseline)}\\ \;+\text{(FTfollow-up—FTbaseline)}\\ \;+\text{(MTfollow-up—MTbaseline)}\\ \Delta \text{D4}-\text{6MFT}=\text{(D4-6Tfollow}-\text{up—D4-6Tbaseline)}\\ \;+\text{(FTfollow-up—FTbaseline)}\\ \;+\;\text{(MTfollow-up—MTbaseline)} \end{array}$$

For categorical variables, chi-square tests were used to test the association of caries experience with the variables studied. The Mann-Whitney *U* test or Kruskal-Wallis H test was employed to study the distribution of decayed, missing, and filled teeth (ΔDMFT) scores according to different variables. For continuous variables, logistic regression was used to test the association of caries experience with the variables, and negative binominal regression was employed to study the distribution of ΔDMFT scores. Independent variables with *p* < 0.50 in the binary analysis were included as covariates in the multivariate analysis.

For the multivariate analyses, the Poisson model, negative binomial model and zero-inflated model were considered to study the relationships between the ΔDMFT scores and the selected variables. Vuong's test was employed to choose an appropriate model. Backward stepwise selection was used to filter the variables in the multivariate regression analysis.

## Results

### General Information

**Figure 2:**
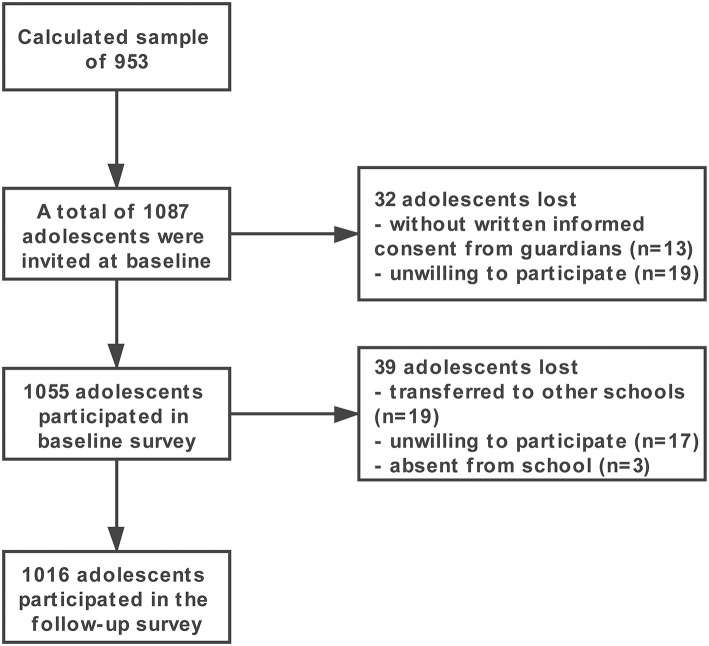
Flowchart of the survey.

A total of 1,087 adolescents were invited, and 1,055 completed the baseline survey. The baseline response rate was 97.06%. A total of 1,016 adolescents completed all evaluations in the longitudinal study (a 96% follow-up rate). Among them, 591 were boys, and 425 were girls. The mean age at baseline was 13.19 ± 0.40 years. Thirty-nine children were lost to follow-up primarily because they transferred schools, were absent from school on the follow-up day, or were unwilling to attend the follow-up examination. Figure 2 displays a flowchart describing the subjects in this study.

The Cronbach's alpha of the questionnaire was 0.75, indicating good reliability. For the inter-examiner reliability of the caries examinations, the Cohen's kappa values were 0.85 (Examiner A vs. B), 0.83 (Examiner B vs. C) and 0.91 (Examiner C vs. A) at baseline. The Cohen's kappa values were 0.80 (Examiner A vs. B), 0.78 (Examiner B vs. C), and 0.86 (Examiner C vs. A) at the 1-year follow-up assessment.

The dentin caries prevalence rate was 36.2%; when enamel caries were included, the rate was 87.8% at baseline. At the 1-year follow-up, the caries prevalence rate was 91.5% (enamel and dentin caries) and 49.6% (dentin caries). The incidence rate for dentin caries was 37.0%. When enamel caries were included, the incidence rate was 47.6%. The mean ΔD1-6MFT was 1.41 ± 2.06, and the mean ΔD4-6MFT was 1.08 ± 1.85. Table 1 shows the profile of caries in the studied population.

**Table 1:** Mean caries experience and severity at tooth and surface levels at baseline and one-year follow-up assessments.

	**D_1−3_** **Mean (SD)**	**D_4−6_** **Mean (SD)**	**D_1−6_** **Mean (SD)**	**M**	**F**
**BASELINE**					
Tooth	3.70 (3.04)	0.62 (1.07)	4.32 (3.28)	0.003 (0.05)	0.10 (0.59)
Surface	4.77 (4.05)	0.68 (1.22)	5.44 (4.38)	0.01 (0.27)	0.10 (0.59)
**1-YEAR FOLLOW-UP**					
Tooth	3.86 (2.84)	1.46 (2.05)	5.32 (3.52)	0.004 (0.06)	0.19 (0.72)
Surface	5.13 (3.65)	2.59 (3.10)	7.72 (4.77)	0.02 (0.31)	0.20 (0.84)

### All Participants

**Figure 3:**
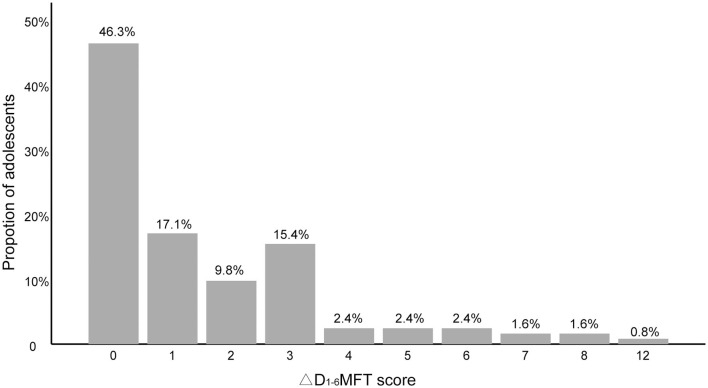
Increase in dentin caries (ΔD4-6MFT) of all participants (*n* = 1,016).

For all participants, the distribution of the ΔD4-6MFT scores was positively skewed, with a skewness of 2.1 (Figure 3). Table 2 shows the results of the binary analysis. Independent variables with *p* < 0.50 in the binary analysis were submitted to further multivariate analysis. As a result, household monthly income and dental flossing were excluded from the model.

**Table 2:** Incidence proportion and rank of median D_4−6_MFT scores among independent variables (*n* = 1,016).

**Independent variables**	***n* (%)**	**Dentin caries**			
		**Incidence proportion *n* (%)**	***p^a^***	**Rank of median D_4−6_MFT score**	***p^b^***
Sex			0.038		0.063
Female	425 (41.8)	173 (40.7)		527	
Male	591 (58.2)	203 (34.3)		495	
Residence			0.360		0.549
Suburban	516 (50.8)	198 (38.4)		503	
Urban	500 (49.2)	178 (35.6)		513	
Caregiver			0.175		0.115
Other people	278 (27.4)	93 (33.5)		521	
Father	111 (10.9)	37 (33.3)		489	
Mother	627 (61.7)	246 (39.2)		486	
Education of caregiver			0.128		0.807
≥9 years	153 (15.1)	65 (42.5)		508	
<9 years	863 (84.9)	311 (36.0)		513	
Household monthly income (CNY)			0.738		0.691
<3,000	238 (23.4)	93 (39.1)		519	
3,000-7,000	616 (60.6)	223 (36.2)		503	
≥7,000	162 (15.9)	60 (37.0)		511	
One-child family			0.011		0.379
No	788 (77.6)	308 (39.1)		522	
Yes	228 (22.4)	68 (29.8)		504	
Frequency of tooth brushing			0.092		0.608
<2 times per day	606 (59.6)	237 (39.1)		512	
≥2 times per day	410 (40.4)	139 (33.9)		503	
Dental flossing			0.576		0.562
No	934 (91.9)	348 (37.3)		510	
Yes	82 (8.1)	28 (34.1)		492	
Toothpaste			0.332		0.237
Non-fluoride	266 (26.2)	105 (39.5)		524	
Fluoride	750 (73.8)	271 (36.1)		502	
Frequency of snack consumption			0.256		0.081
≥1 per day	696 (68.5)	269 (38.6)		517	
Not every day	320 (31.5)	107 (33.4)		488	
Frequency of sweet drink consumption			0.268		0.841
≥1 per day	365 (35.9)	138 (37.8)		510	
Not every day	651 (64.1)	238 (36.6)		507	
Orthodontics appliance			0.706		0.639
Yes	24 (2.4)	8 (33.3)		509	
No	992 (97.6)	368 (37.1)		484	
Dental attendance in the past 6 months			0.125		0.121
Yes	560 (55.1)	219 (39.1)		519	
No	456 (44.9)	157 (34.4)		494	
Dental insurance			0.478		0.359
Yes	234 (23.0)	82 (35.0)		495	
No	782 (77.0)	294 (37.6)		512	
Saliva secretion (ml/min)			0.300		0.279
<0.25	234 (27.4)	110 (39.6)		522	
≥0.25	782 (72.6)	266 (36.0)		503	
Saliva buffering capability (pH)			0.121		0.197
<3.5	321 (31.6)	132 (41.1)		526	
3.5–4.25	300 (29.5)	111 (37.0)		509	
≥4.25	395 (38.9)	133 (33.7)		492	
Past caries experience			<0.001		<0.001
Baseline D_4−6_MFT = 0	648 (63.8)	204 (31.5)		481	
Baseline D_4−6_MFT > 0	368 (36.2)	172 (46.7)		556	
	***Mean (SD)***	***OR^c^***	***p^c^***	***OR^d^***	***p^d^***
Plaque index	1.31 (0.68)	0.877	0.179	1.011	0.873
Cariostat score	1.78 (0.67)	0.752	0.003	1.228	0.011
Baseline age (years)	13.19 (0.40)	1.032	0.849	0.774	0.250

a *Chi-squared test*.

b *Mann-Whitney U test or Kruskal-Wallis H test*.

c *Logistic regression*.

d *Negative binomial regression*.

The result of Vuong's test showed that the zero-inflated negative binomial (ZINB) model was the best model to fit the data (*p* < 0.001). After the multivariate analysis, variables with a *P* < 0.05 were retained in the models. The ΔD4-6MFT score was related to sex (*p* = 0.001, IRR = 0.732, 95% CI: 0.663–0.945) and cariostat score (*p* = 0.012, IRR = 1.182, 95% CI: 1.037–1.347). In the zero-inflated part, individuals with past dentin caries experience had an increased likelihood of having an increment in D4-6MFT score (*p* < 0.001, OR = 1.765, 95% CI: 1.343–2.321) (Table 3).

**Table 3:** Dentin caries risk factors of all participants.

**Negative binomial portion (ΔD_4−6_MFT > 0)**	**Variables**	**IRR**	**95% CI**	***p***
	**Cariostat score**	1.182	1.037–1.347	0.012
	**Sex**			
	Male	0.732	0.663–0.945	0.001
	Female^*^	–	–	–
Zero-inflated portion (ΔD_4−6_MFT = 0)	Variables	OR	95% CI	*p*
	**Past caries experience**			
	Baseline D_3−6_MFT > 0	1.765	1.343–2.321	<0.001
	Baseline D_3−6_MFT = 0^*^	–	–	–

* *Reference group*.

### Caries-Free Group

There were 129 individuals who were caries free (D1-6MFT = 0) at baseline. After 1 year, 6 patients missed the follow-up assessment. Among the remaining 123 individuals, 57 (46.3%) adolescents were still caries free. The 123 individuals were categorized as the caries-free group. The mean ΔD1-6MFT was 1.54 ± 2.12, and the mean ΔD4-6MFT was 0.07 ± 0.43 (ranging from 0 to 3). Only four individuals developed dentin caries; thus, the enamel caries and dentin caries were both included in the analysis of the caries-free group, where the ΔD1-6MFT score was treated as the dependent variable.

**Figure 4:**
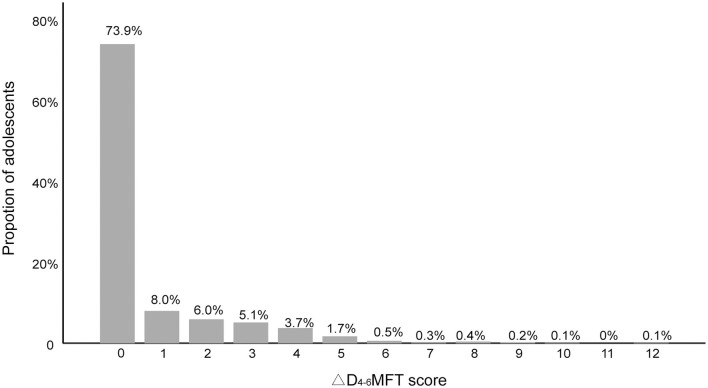
Increase in enamel and dentin caries (ΔD1-6MFT) of caries-free group (*n* = 123).

Figure 4 shows the distribution of the ΔD1-6MFT score of the caries-free population, which was positively skewed, with a skewness of 2.00. As in the analysis of all participants, the caregivers, household monthly income, frequency of toothbrushing and orthodontic appliances were excluded after the binary analysis yielded *p*-values over 0.5 for these variables in all binary tests (Table 4).

**Table 4:** Incidence proportion and rank of median D_1−6_MFT scores among independent variables (*n* = 123).

**Independent variables**	***n* (%)**	**Enamel and dentin caries**			
		**Incidence proportion (%)**	***p^a^***	**Rank of median D_1−6_MFT score**	***p^b^***
Sex			0.001		0.001
Female	35 (28.5)	27 (77.1)		76	
Male	88 (71.5)	39 (44.3)		56	
Residence			0.243		0.245
Suburban	50 (40.7)	30 (60.0)		66	
Urban	73 (59.3)	36 (49.3)		59	
Caregiver			0.952		0.836
Other people	31 (61.8)	41 (53.9)		59	
Father	16 (13.0)	9 (56.3)		62	
Mother	76 (25.2)	16 (51.6)		65	
Education of caregiver			0.396		0.398
≥9 years	18 (14.6)	71 (46.4)		56	
<9 years	105 (85.4)	413 (47.9)		63	
Household monthly income (CNY)			0.905		0.826
<3,000	18 (14.6)	9 (50.0)		62	
3,000–7,000	78 (63.4)	43 (55.1)		63	
≥7,000	27 (22.0)	14 (51.9)		58	
One-child family			0.261		0.262
No	86 (69.9)	49 (57.0)		64	
Yes	37 (30.1)	17 (45.9)		57	
Frequency of tooth brushing			0.972		0.972
<2 times per day	71 (57.7)	38 (47.2)		62	
≥2 times per day	52 (42.3)	28 (48.3)		62	
Dental flossing			0.972		0.343
No	111 (90.2)	449 (53.5)		70	
Yes	12 (9.8)	35 (43.8)		61	
Toothpaste			0.275		0.277
Non-fluoride	29 (23.6)	13 (44.8)		63	
Fluoride	94 (76.4)	53 (56.4)		56	
Frequency of snack consumption			0.055		0.022
≥1 per day	48 (39.0)	32 (66.7)		76	
Not every day	75 (61.0)	34 (45.3)		55	
Frequency of sweet drink consumption			0.320		0.416
≥1 per day	80 (65.0)	44 (55.0)		74	
Not every day	43 (35.0)	22 (51.2)		64	
Orthodontics appliance			0.647		0.649
Yes	3 (2.4)	2 (66.7)		70	
No	120 (97.6)	64 (53.3)		62	
Dental attendance in the past 6 months			0.986		0.986
Yes	56 (54.5)	30 (53.6)		62	
No	67 (45.5)	36 (53.7)		62	
Dental insurance			0.090		0.216
Yes	23 (81.3)	16 (69.6)		60	
No	100 (18.7)	50 (50.0)		70	
Saliva secretion (ml/min)			0.754		0.272
<0.25	35 (28.5)	18 (51.4)		64	
≥0.25	88 (71.5)	48 (54.5)		57	
Saliva buffering capability (pH)			0.684		0.175
<3.5	40 (32.5)	23 (57.5)		64	
3.5–4.25	26 (21.1)	14 (53.8)		62	
≥4.25	57 (46.3)	29 (50.9)		61	
	***Mean(SD)***	***OR^c^***	***p^c^***	***OR^d^***	***p^d^***
Plaque index	1.13 (0.66)	0.877	0.179	1.011	0.873
Cariostat score	1.46 (0.59)	0.752	0.003	1.228	0.011
Baseline age (years)	13.12 (0.40)	1.032	0.849	0.774	0.250

a *Chi-squared test*.

b *Mann-Whitney U test or Kruskal-Wallis H test*.

c *Logistic regression*.

d *Negative binominal regression*.

For the multivariate analysis, the result of Vuong's test showed that the ZINB model was also the best model to fit the data (*P* = 0.049). In the zero-inflated portion, the result shows that male individuals (*p* = 0.001 OR = 0.223, 95% CI: 0.089–0.558) and individuals who ate snacks not every day (*p* = 0.013 OR = 0.387, 95% CI: 0.175–0.853) had a decreased likelihood of having an increment in D1-6MFT score (Table 5). The ΔD1-6MFT score was related to belonging to a one-child family (*p* = 0.030, IRR = 0.606, 95% CI: 0.385–0.954) and the use of fluoride toothpaste (*p* = 0.047, IRR = 0.632, 95% CI: 0.401–0.995). In addition, compared with individuals with a low saliva buffering capability (pH < 3.5), individuals with a high saliva buffering capability (pH ≥ 4.25) had a low ΔD1-6MFT score (*p* = 0.025, IRR = 0.563, 95% CI: 0.341–0.931).

**Table 5:** Enamel and dentin caries risk factors of caries-free group.

**Negative binomial portion (ΔD_1−6_MFT > 0)**	**Variables**	**IRR**	**95% CI**	***p***
**ONE-CHILD FAMILY**				
	Yes	0.606	0.385–0.954	0.030
	No^*^	–	–	–
**TOOTHPASTE**				
	Fluoride	0.632	0.401–0.995	0.047
	Non-fluoride^*^	–	–	–
**SALIVA BUFFERING CAPABILITY (pH)**				
	≥4.25	0.563	0.341–0.931	0.025
	3.5−4.25	0.843	0.528–1.437	0.475
	<3.5^*^	–	–	–
Zero-inflated portion (ΔD_1−6_MFT = 0)	Variables	OR	95% CI	*p*
	**Sex**			
	Male	0.223	0.089–0.558	0.001
	Female^*^	–	–	–
**FREQUENCY OF SNACK CONSUMPTION**				
	Not every day	0.387	0.175–0.853	0.019
	≥1 per day^*^	–	–	–

* *Reference group*.

## Discussion

With the broadening of understanding and the definition of dental caries, the focus of caries detection, diagnosis, and management has shifted from advanced caries to initial caries (29); thus, the merged ICDAS criteria were employed in this study. ICDAS was designed to identify the clinical stages of the caries process (22). In the present study, enamel caries and dentin caries were both evaluated. For all participants, only the increase in dentin caries was considered, mainly for two reasons. First, most adolescents in this study had at least one enamel caries lesion at baseline, and only 8.5% children were “truly caries free” at the 1-year follow-up assessment; hence it would be meaningless to investigate enamel caries risk factors in the whole population. In addition, to allow for comparison with the results of other studies that have used the World Health Organization WHO criteria, the D4-6 threshold for caries, which is considered closest to the WHO criteria, was employed (30). The increase in enamel caries was analyzed in the caries-free population.

The DMFT index is the count of decayed, missing and filled teeth and it is commonly used in epidemiological surveys. The DMFT count is often overdispersed, with excess zero counts. To address this type of distribution, Lewsey and Thomson applied ZINB regression models to fit the data (31). In the present study, Vuong's test showed that the ZINB regression model fit the data of this study better than other common statistical models. Thus, ZINB regression analysis was employed to analyse the effects of the independent variables.

The results showed that the higher the individual cariostat score is, the higher the possible ΔD4-6MFT score he or she could have. Developed by Shimono, the cariostat test is a colorimetric test that determines the acidogenecity of oral microorganisms in plaque through changes at a pH of 7 (32). The color changes from blue (scores 0, pH of 6.1 ± 0.3) to yellow (scores 3.0, pH of 4.0 ± 0.3). Cariostat has the ability to determine the acidogenecity of the bacteria without being affected by the amount of the microorganism colony (32). The higher the cariostat score, the higher the acidogenecity of the oral microorganisms. Therefore, the result found in the present study reveals a significant relationship between the composition of biofilm colonized on tooth surfaces and the increase in caries. Biofilm with active acidproductive and aciduric bacteria is a risk factor for dentin caries, which is consistent with previous studies (33). Cariostat has been proven to be a valid caries predictor in children (25). Unlike other chair-side cariogenic microbiology tests, such as Dentocult SM or Dentocult LB (which are no longer produced), cariostats assess the bacteria in dental plaques rather than in saliva. This results in increased accuracy because cariogenic bacteria work in the form of plaque on tooth surfaces.

Nevertheless, the results showed that the cariostat score was not a predictor for enamel caries when analyzing the caries free group, where most individuals developed only enamel caries. This result could be because the early stage of caries is less affected by bacteria with comparatively low acid-producing capability, whereas the cariostat test works by testing the activity of acid-producing bacteria in the biofilm (34). Hence, a potential limitation of the cariostat test is that it does not show good prediction performance in the early stage of caries.

Another risk factor identified in the whole population was sex; the results showed that females were more likely to have higher ΔD4-6MFT scores than males. In addition, in the caries-free population, females were more likely to develop new caries than males. A similar phenomenon has been observed in some other populations (35). The reason for the sex difference in caries development was inconclusive. The different salivary composition and flow rate, dietary habits, genetic variations, early eruption of teeth, and particular social roles in families were the most common factors explaining the consistent trend of higher caries rates in females than in males (36, 37).

It is not surprising to find that past caries experience was the only risk factor for the onset of new dentin caries in the whole population. As observed in other populations (38, 39), past caries experience was the strongest predictor of new dentin caries. However, it is not an ideal approach, as it is not conducive to the early prevention of dental caries. A 2-years cohort study reported not only that moderate and severe caries were associated with caries incidence but also that the initial caries were associated with caries incidence (40). To determine the initial factors for caries onset, only individuals whose D1-6MFT score was “0” were included in the further analysis.

Our results showed that in addition to sex, the frequency of snack consumption was another risk factor for the onset of enamel and dentin caries (mostly enamel caries) in the caries-free population. Snack consumption has gained an increasing role as a risk indicator for caries development in children (41, 42); even non-sweet snacks are potentially cariogenic due to their content of extensively hydrolysed starch (43). In the present study, individuals eating snacks every day had a higher risk of caries because it extends the duration of acid production and exposure, thereby tipping the scale toward the development of caries (44). Diet has been recognized as a caries risk factor in various models explaining the etiology of dental caries (45–47). Recently, Meyer-Lueckel et al. introduced a pathogenesis model of caries based on the ecological plaque hypothesis, where a greater role has been assigned to sugar in the etiology (48). They hold the view that caries is a disease of civilizations that consume a greater amount of sugar, which was not the case throughout most of human history (49). Furthermore, the frequent consumption of fermentable carbohydrates causes a pathological shift in the oral microflora, leading to an increase in cariogenic bacteria (50). Therefore, it is meaningful to advocate less snack consumption in adolescents to prevent dental caries.

In the negative binomial portion, individuals from one-child families or who used fluoride toothpaste were likely to have relatively low ΔD1-6MFT scores. These results were consistent with those in previous studies (35, 51). Fluoride can help the hydroxyapatite remineralization process, and it is most abundant in the outer layers of enamel, which explains why individuals using fluoride toothpaste were likely to have less enamel caries (52). Currently, there is no fluoride tap water in mainland China, and for most places like Foshan in South China, the water fluoride concentration was low (<0.30 mg/L) (53). As a result, it is critical to promote clinical and personal fluoride usage among children. Additionally, in this study, individuals with a high saliva buffering capability (pH ≥ 4.25) were likely to have a lower ΔD1-6MFT score than those with a low saliva buffering capability (pH < 3.5), implicating of saliva buffering tests to distinguish risk adolescents. Saliva buffering fast check kits, are now provided by some manufacturers, like the “Saliva-check” by GC, and “CRT buffer” by Ivoclar. These kits can quickly test the saliva buffering capability and easily be used by non-professional practitioners. These kits make it possible to to check the saliva buffering capability for both clinical and public health use in future.

An interesting finding in this study is that the variables regarding oral hygiene, including the plaque index and teeth brushing frequency, were found to be negatively correlated with caries development. Oral hygiene was not a caries risk factor in this population. The failure of oral hygiene in prediction among this population could due to their genetic background. Strömberg et al. indicated that individuals with a specific genetic background may develop more caries from bad oral hygiene than individuals with other genetic backgrounds (54). Hence, variables such as PlI and tooth brushing frequency could still be predictable in other populations with different genetic backgrounds.

Adolescence is a vital life stage where children begin to develop self-performed oral health habits instead of relying solely on parental supervision. Ostberg et al. reported that children's oral health-related behaviors will change significantly during adolescence (6). Adolescents at baseline were all at their first year in middle school, when they started to “gain more freedom” in oral health behaviors (e.g., less brushing teeth or eating more snacks without parent's supervision). Furthermore, evidences from other longitudinal research indicate a significantly high rate of caries progression in 11–16-year-olds (45, 46). As a result, though remained free of caries until adolescence, it is still possible for individuals in caries free group to developed dental caries lesions in 1-year follow up. However, in the present study X-ray was not applied in the caries detection, and some proximal initial caries could have been missed. As a result, the results of the “caries-free group” still need to be seen with caution.

The present evidence shows that dental caries, when including enamel caries, could be very prevalent with a prevalence rate of over 80% among most populations aged 12–15 years, as was our population (55–57). The reason for a high prevalence rate could be insufficient awareness of initial caries in most adolescents, their guardians and even clinical dentists. It was suggested that approximately half of the initial proximal lesions in 15-year-old adolescents progress to cavitated lesions at the age of 20 (58). Preventing the onset and development of enamel caries deserves more attention. According to the International Caries Classification and Management System (ICCMS™), the caries activity judgement is a practical tool in clinical management of caries lesions (59). The treatment decision routine with lesion activity judgement has been proven reliable in daily clinical practice. Additionally, the unique potential of activity judgement makes it a research tool for exploring transition patterns of caries lesions in response to different interventions. Most valuably, caries activity judgement in the planning and organization of dental public health services can provide information about the amount of active non-cavitated lesions indicated for non-operative treatment (60). Nevertheless, the information about lesion activities was not recorded in our data, which could be a weakness of this study. The main reason why caries lesion activities were not evaluated is that the lesion activity could change with time, and the activity of some enamel caries could change without substantial progression. The change could be mercurial, under which circumstance a shorter follow-up interval (usually 3 months or shorter) should be set. The present study design was not able to monitor the activity change throughout the year. Future studies with a shorter follow-up interval should be conducted to explore the transition patterns of caries lesions.

In many developed countries, the level of dental caries experience has declined in the past few decades (61). The incidence density was estimated as 0.5–0.7 per year per person (count by D3MFT, represented as D4-6MFT in this study) in an ecological time-trend study in adolescents aged 12–18 years in Norway (62). The incidence density of 2,848 adolescents in another 4-years cohort study in Sweden was estimated as 0.5 per year per person (count by D4-6S) (63). In the present study, the incidence density was 1.08 (count by D4-6MFT). This number should be higher than those in adolescents in Norway and Sweden. Although the caries prevalence rate in China is lower than that in developed countries (1), it shows a growing trend, and the incidence is also higher than those in developed countries. The observed high incidence of caries could mainly be the result of inadequate awareness of public health in China. Benefitting from rapid economic development, children are consuming more sugars than 10 years ago; nevertheless, few dental public health measures have been applied, whereas they have been applied for decades in developed countries. Therefore, in China more public health resources must be devoted to caries.

The increase in caries in the studied adolescents was higher than those in developed countries, indicating more that dental public health resources are necessary. Our results suggest that past caries experience is still the strongest predicator for dentin caries. Female individuals and individuals with a high cariostat score tended to develop more dentin caries. These factors are suitable for distinguishing high-risk individuals. After excluding the influence of past caries experience, the frequency of snack consumption, sex, saliva buffering capability, fluoride toothpaste usage, and belonging to a one-child family were risk factors of dental caries. As a result, advocating less snack consumption and promoting fluoride toothpaste could help to prevent caries; meanwhile, sex, saliva buffering capability, and number of children in the family should be indicators for distinguishing the high-risk individuals. However, the result should be treated with caution because no X-ray was applied in the caries detection; thus, the proximal initial caries could have been missed, and a multistage cluster sampling technique was adopted, which could amplify the sampling error. These limitations could cause some bias in the result.

## Data Availability Statement

The datasets generated for this study are available on request to the corresponding author.

## Ethics Statement

The studies involving human participants were reviewed and approved by Ethical Review Committee of Guanghua School of Stomatology, Sun Yat-Sen University. Written informed consent to participate in this study was provided by the participants' legal guardian/next of kin.

## Author Contributions

HL substantially contributed to conception and design of this study. KW, LP, CF, TC, and LY contributed to acquisition, analysis, and interpretation of data. KW drafted the manuscript. LP, CF, TC, LY, and HL revised the manuscript for important intellectual content critically. All authors gave final approval to this article, and all agreed to be accountable for all aspects of the work in ensuring that questions relating to the accuracy or integrity of any part of the work are appropriately investigated and resolved.

## Conflict of Interest

The authors declare that the research was conducted in the absence of any commercial or financial relationships that could be construed as a potential conflict of interest.
