# Supplementary material for: Enamel and Dentin Caries Risk Factors of Adolescents in the Context of the International Caries Detection and Assessment System (ICDAS): A Longitudinal Study
Source: Front Pediatr. 2020 Jul 28;8:419. doi: 10.3389/fped.2020.00419 (PMC7399199; doi:10.3389/fped.2020.00419)
Supplement: Supplementary file 1 [file Table_1.DOCX]

STROBE Statement—checklist of items that should be included in reports of observational studies

|  | Item No. | Recommendation | Page  No. | Relevant text from manuscript |
| --- | --- | --- | --- | --- |
| **Title and abstract** | 1 | (*a*) Indicate the study’s design with a commonly used term in the title or the abstract | 1 | A longitudinal study |
|  |  | (*b*) Provide in the abstract an informative and balanced summary of what was done and what was found | 1 | This one-year longitudinal study was conducted in 2018 and 2019; 13- to 14-year-old adolescents were recruited.  The ZINB analysis found that individuals with caries at baseline were more likely to develop new dentin caries. |
| Introduction | | | |  |
| Background/rationale | 2 | Explain the scientific background and rationale for the investigation being reported | 2 | The global caries prevalence was approximately 50% among 12-15-year-old adolescents and over 70% among 17-year-old adolescents. In China, the prevalence of caries among 12-year-old children has increased from 28.9% to 38.5% in the last decade. |
| Objectives | 3 | State specific objectives, including any prespecified hypotheses | 2 | the primary objective of this study was to investigate the caries risk factors in adolescents in South China by conducting a one-year longitudinal study, specifying the risk factors without the affection of past caries experience |
| Methods | | | |  |
| Study design | 4 | Present key elements of study design early in the paper |  | Not mentioned |
| Setting | 5 | Describe the setting, locations, and relevant dates, including periods of recruitment, exposure, follow-up, and data collection | 2 | The baseline data were collected from March to April 2018 in Foshan. Foshan is a medium-sized city in Guangdong Province in southern China, with a population of 7.6 million. The gross domestic product (GDP) was CNY 124,324 (USD 18,018) per capita in 2017. The water fluoride concentration is 0.16 mg/L. |
| Participants | 6 | (*a*) *Cohort study*—Give the eligibility criteria, and the sources and methods of selection of participants. Describe methods of follow-up  *Case-control study*—Give the eligibility criteria, and the sources and methods of case ascertainment and control selection. Give the rationale for the choice of cases and controls  *Cross-sectional study*—Give the eligibility criteria, and the sources and methods of selection of participants | 3 | All the students in these classes whose parents were Han Chinese and permanent residents in Foshan were recruited for this study. Those who reported systematic illness or antibiotic use in the preceding 2 weeks were excluded. |
|  |  | (*b*) *Cohort study*—For matched studies, give matching criteria and number of exposed and unexposed  *Case-control study*—For matched studies, give matching criteria and the number of controls per case |  | Not applicable |
| Variables | 7 | Clearly define all outcomes, exposures, predictors, potential confounders, and effect modifiers. Give diagnostic criteria, if applicable | 3 | The merged International Caries Detection and Assessment System (ICDAS) criteria were used. |
| Data sources/ measurement | 8* | For each variable of interest, give sources of data and details of methods of assessment (measurement). Describe comparability of assessment methods if there is more than one group | 4 & 5 | Decayed teeth were recorded as enamel caries (coded 1-3 in the ICDAS system, D1-3) or dentin caries (coded 4-6 in the ICDAS system, D4-6). Filled surfaces and missing teeth due to caries (the reasons for missing teeth were obtained from the questionnaire) were also recorded.  △D1-6MFT = (D4-6Tfollow-up - D4-6Tbaseline) + (D1-3Tfollow-up - D1-3Tbaseline) + (FTfollow-up - FTbaseline) + (MTfollow-up - MTbaseline)  △D4-6MFT = (D4-6Tfollow-up - D4-6Tbaseline) + (FTfollow-up - FTbaseline) + (MTfollow-up - MTbaseline) |
| Bias | 9 | Describe any efforts to address potential sources of bias | 3 & 4 | Three examiners took a 12-hour e-learning course on ICDAS before the study. To calculate the consistency of the inter-examiner reliability, 105 subjects were randomly chosen during the oral examination, they were re-examined by another examiner right after examined by the first examiner (35 examinee from A to B, 35 examinee from B to C, and 35 examinee from C to A).  Similar to the baseline assessment, 105 subjects were randomly re-examined to calculate Cohen’s kappa. |
| Study size | 10 | Explain how the study size was arrived at | 2 | PASS 11.0.7 (NCSS, LLC) was applied to calculate the sample size. The Poisson Regression Model was used. The significance level was set at 0.05, with a power of 0.8, assuming a 20% nonresponse rate; thus, the total sample size was calculated as at least 953 participants. |

Continued on next page

| Quantitative variables | 11 | Explain how quantitative variables were handled in the analyses. If applicable, describe which groupings were chosen and why | 3 & 4 | The compulsory education is 9 years in China, so ‘9 years’ was set as a threshold to distinguish the education of the caregivers in the variable ‘education of caregiver’. As to the categories in the variable ‘household monthly incoming’, according to Foshan government, households with the lowest 20% annual average household income is classified into low income group, while households with the highest 20% annual average household income is classified into high income group. In 2017, the threshold for low income group was 35339.38 CNY (about 3000 CNY per month), and 85774.21 CNY (about 7000 CNY per month) for high income group in Foshan.  The ﬂow rate was recorded into ‘abnormal (<0.25)’ or ‘normal (≥0.25)’.  The buffering capability was recorded into ‘low’ (<3.5), ‘moderate’ (3.5-4.25) or ‘high’ (≥4.25). | |
| --- | --- | --- | --- | --- | --- |
| Statistical methods | 12 | (*a*) Describe all statistical methods, including those used to control for confounding | 5 | For categorical variables, chi-square tests were used to test the association of caries experience with the variables studied. The Mann-Whitney U test or Kruskal-Wallis H test was employed to study the distribution of decayed, missing, and filled teeth (∆DMFT) scores according to different variables. For continuous variables, logistic regression was used to test the association of caries experience with the variables, and negative binominal regression was employed to study the distribution of ∆DMFT scores.  For the multivariate analyses, the Poisson model, negative binomial model and zero-inflated model were considered to study the relationships between the ∆DMFT scores and the selected variables. Vuong’s test was employed to choose an appropriate model. | |
|  |  | (*b*) Describe any methods used to examine subgroups and interactions | 6 | There were 129 individuals who were caries free (D1-6MFT=0) at baseline. After one year, 6 patients missed the follow-up assessment. Among the remaining 123 individuals, 57 (46.3%) adolescents were still caries free. The 123 individuals were categorized as the caries-free group. | |
|  |  | (*c*) Explain how missing data were addressed | 5 | The data of these 1016 adolescents were submitted to further analysis. | |
|  |  | (*d*) *Cohort study*—If applicable, explain how loss to follow-up was addressed  *Case-control study*—If applicable, explain how matching of cases and controls was addressed  *Cross-sectional study*—If applicable, describe analytical methods taking account of sampling strategy |  | Not mentioned | |
|  |  | (*e*) Describe any sensitivity analyses |  | Not applicable | |
| Results | | | | |  |
| Participants | 13* | (a) Report numbers of individuals at each stage of study—eg numbers potentially eligible, examined for eligibility, confirmed eligible, included in the study, completing follow-up, and analysed | 5 | A total of 1087 adolescents were invited, and 1055 of them finished the baseline survey. The baseline response rate was 97.06%. A total of 1016 adolescents finished all evaluations in the longitudinal study (a 96% follow-up rate). The data of these 1016 adolescents were submitted to further analysis. (a 96% follow-up rate) | |
|  |  | (b) Give reasons for non-participation at each stage | 5 | Thirty-nine children were lost to follow-up, primarily because they transferred schools, were absent from school on the follow-up day, or were unwilling to attend the follow-up examination. | |
|  |  | (c) Consider use of a flow diagram |  | Figure 2 | |
| Descriptive data | 14* | (a) Give characteristics of study participants (eg demographic, clinical, social) and information on exposures and potential confounders | 5 | Among them, 591 were boys and 425 were girls. The mean age at baseline was 13.19±0.40 years. | |
|  |  | (b) Indicate number of participants with missing data for each variable of interest |  | Not mentioned | |
|  |  | (c) *Cohort study*—Summarise follow-up time (eg, average and total amount) |  | Not applicable | |
| Outcome data | 15* | *Cohort study*—Report numbers of outcome events or summary measures over time | 5 | The dentin caries prevalence rate was 36.2%, and when enamel caries were included, the rate was 87.8% at baseline. At the one-year follow-up, the caries prevalence rate was 91.5% (enamel and dentin caries) and 49.6% (dentin caries). The incidence rate for dentin caries was 37.0%. When enamel caries were included, the incidence rate was 47.6%. | |
|  |  | *Case-control study—*Report numbers in each exposure category, or summary measures of exposure |  | Not applicable | |
|  |  | *Cross-sectional study—*Report numbers of outcome events or summary measures |  | Not applicable | |
| Main results | 16 | (*a*) Give unadjusted estimates and, if applicable, confounder-adjusted estimates and their precision (eg, 95% confidence interval). Make clear which confounders were adjusted for and why they were included |  | Not applicable | |
|  |  | (*b*) Report category boundaries when continuous variables were categorized |  | Not applicable | |
|  |  | (*c*) If relevant, consider translating estimates of relative risk into absolute risk for a meaningful time period |  | Not applicable | |

Continued on next page

| Other analyses | 17 | Report other analyses done—eg analyses of subgroups and interactions, and sensitivity analyses | 6 | Figure 4 shows the distribution of the ∆D1-6MFT score of the caries-free population, which was positively skewed, with a skewness of 2.00. As in the analysis of all participants, caregivers, household monthly income, frequency of toothbrushing and orthodontic appliances were excluded after the binary analysis cause the P-value of these variables were over 0.5 in all binary tests (Table 4).  For the multivariate analysis, the result of Vuong’s test showed that the ZINB model was also the best model to fit the data (P=0.049). In the zero-inflated portion, the result shows that male individuals (P=0.001 OR=0.223, 95% CI: 0.089-0.558), and individuals who ate snacks less than once per day (P=0.013 OR=0.387, 95% CI: 0.175-0.853) had a decreased likelihood of developing new caries lesions (Table 5). The ∆D1-6MFT score was related to belonging to a one-child family (P=0.030, IRR=0.606, 95% CI: 0.385-0.954) and the use of fluoride toothpaste (P=0.047, IRR=0.632, 95% CI: 0.401-0.995). In addition, compared to individuals with a low saliva buffering capability (pH<3.5), individuals with a high saliva buffering capability (pH≥4.25) had a low △D1-6MFT score (P=0.025, IRR=0.563, 95% CI: 0.341-0.931). | |
| --- | --- | --- | --- | --- | --- |
| Discussion | | | | |  |
| Key results | 18 | Summarise key results with reference to study objectives | 9 | The caries increment in studied adolescents was higher than those in developed countries, indicating more dental public health recourses have to be devoted. Our result suggests that past caries experience is still the strongest predicator for dentin caries. Female individuals, and individuals with a high cariostat score were tend to develop more dentin caries. These factors are suitable to applied to distinguish the high risk individuals. After excluding the influence of past caries experience, the frequency of snack consumption, sex, saliva buffering capability, fluoride toothpaste usage and belonging to a one-child family were risk factors of dental caries. | |
| Limitations | 19 | Discuss limitations of the study, taking into account sources of potential bias or imprecision. Discuss both direction and magnitude of any potential bias | 7 | Before taking about the findings, some limitations of the study must be considered. First, the study adopted a multistage cluster sampling technique, which could amplify the sampling error. Second, the DMFT score, which taken all fillings into consideration was used as the index in the analysis, and that could overestimate the number of caries. | |
| Interpretation | 20 | Give a cautious overall interpretation of results considering objectives, limitations, multiplicity of analyses, results from similar studies, and other relevant evidence | 9 | As a result, advocating less snack consumption and promotion of fluoride toothpaste could help to prevent caries, while sex, saliva buffering capability and family children should be indicators to distinguish the high risk individuals. However, the result excluding the influence of past caries experience should be treated with caution cause no X-ray was applied in the caries detection and the proximal initial caries could be neglected, which could cause bias on the result. | |
| Generalisability | 21 | Discuss the generalisability (external validity) of the study results |  | Not mentioned | |
| Other information | |  | | |  |
| Funding | 22 | Give the source of funding and the role of the funders for the present study and, if applicable, for the original study on which the present article is based | 10 | The present study was supported by the National Natural Science Foundation of China (no. 81570967) and China’s Forth National Oral Health Epidemiology Survey (no. 201502002). | |

*Give information separately for cases and controls in case-control studies and, if applicable, for exposed and unexposed groups in cohort and cross-sectional studies.

**Note:** An Explanation and Elaboration article discusses each checklist item and gives methodological background and published examples of transparent reporting. The STROBE checklist is best used in conjunction with this article (freely available on the Web sites of PLoS Medicine at http://www.plosmedicine.org/, Annals of Internal Medicine at http://www.annals.org/, and Epidemiology at http://www.epidem.com/). Information on the STROBE Initiative is available at www.strobe-statement.org.
